# Machine learning-based prediction of sertraline concentration in patients with depression through therapeutic drug monitoring

**DOI:** 10.3389/fphar.2024.1289673

**Published:** 2024-03-06

**Authors:** Ran Fu, Xin Hao, Jing Yu, Donghan Wang, Jinyuan Zhang, Ze Yu, Fei Gao, Chunhua Zhou

**Affiliations:** ^1^ Department of Clinical Pharmacy, The First Hospital of Hebei Medical University, Shijiazhuang, China; ^2^ The Technology Innovation Center for Artificial Intelligence in Clinical Pharmacy of Hebei Province, The First Hospital of Hebei Medical University, Shijiazhuang, China; ^3^ Dalian Medicinovo Technology Co., Ltd, Dalian, China; ^4^ Beijing Medicinovo Technology Co., Ltd, Beijing, China; ^5^ Institute of Interdisciplinary Integrative Medicine Research, Shanghai University of Traditional Chinese Medicine, Shanghai, China

**Keywords:** sertraline, concentrations, therapeutic drug monitoring, machine learning, XGBoost

## Abstract

**Background:** Sertraline is a commonly employed antidepressant in clinical practice. In order to control the plasma concentration of sertraline within the therapeutic window to achieve the best effect and avoid adverse reactions, a personalized model to predict sertraline concentration is necessary.

**Aims:** This study aimed to establish a personalized medication model for patients with depression receiving sertraline based on machine learning to provide a reference for clinicians to formulate drug regimens.

**Methods:** A total of 415 patients with 496 samples of sertraline concentration from December 2019 to July 2022 at the First Hospital of Hebei Medical University were collected as the dataset. Nine different algorithms, namely, XGBoost, LightGBM, CatBoost, random forest, GBDT, SVM, lasso regression, ANN, and TabNet, were used for modeling to compare the model abilities to predict sertraline concentration.

**Results:** XGBoost was chosen to establish the personalized medication model with the best performance (*R* ^2^ = 0.63). Five important variables, namely, sertraline dose, alanine transaminase, aspartate transaminase, uric acid, and sex, were shown to be correlated with sertraline concentration. The model prediction accuracy of sertraline concentration in the therapeutic window was 62.5%.

**Conclusion:** In conclusion, the personalized medication model of sertraline for patients with depression based on XGBoost had good predictive ability, which provides guidance for clinicians in proposing an optimal medication regimen.

## Introduction

Depression is a common illness that causes dysfunction in various spheres of individual and social life, severely limits psychosocial functioning, and diminishes quality of life (Malhi and Mann, 2018). Currently, depression is considered the fourth leading cause of global disease burden (Farnam et al., 2017). Depression rates in young people have risen sharply in the past decade, and females have a greater incidence of elevated depressive symptoms than males (Shorey et al., 2022; Thapar et al., 2022). Thus, reasonable treatment for depression is a priority.

Sertraline, a kind of selective serotonin reuptake inhibitor (SSRI) antidepressant, exerts antidepressant effects by inhibiting the reuptake of 5-hydroxytryptamine (5-HT) in central neurons and is often used as a first-line treatment for depression (Shorey et al., 2022; Thapar et al., 2022). It has good efficacy in treating various forms of depression, such as psychotic depression and major depressive disorder-related postnatal depression (Milgrom et al., 2015; Kamijima et al., 2018; Flint et al., 2021). Sertraline is metabolized mainly by CYP2B6 and CYP2C19 in the liver, where it generates the metabolite N-desmethylsertraline with lower activity. Sertraline and its metabolites are mainly excreted in feces and urine. During clinical usage, drug-related adverse reactions, such as corrected QT interval prolongation, bleeding, sexual dysfunctions, inflammation, or fracture risk (Kubanek et al., 2021), may occur, which may be related to the dosage and plasma concentration of sertraline. Due to the large individual differences in sertraline concentrations, therapeutic drug monitoring (TDM) is necessary to maintain the plasma concentration within the therapeutic window. Based on empirical evidence, the Arbeitsgemeinschaft für Neuropsychopharmakologie und Pharmakopsychiatrie (AGNP) consensus guidelines define the level of recommendation to use TDM of sertraline as level 2: recommended, with a therapeutic reference range of 10–150 ng/mL (Hiemke et al., 2018-01).

Machine learning (ML), deep learning (DL), and swarm intelligence (SI) are emerging artificial intelligence (AI) techniques that have recently been applied in medical research. By processing high-volume data, they can evaluate data-driven estimations from multiple variables and capture non-linear variable relations to achieve high accuracy in predicting clinical outcomes (Guo et al., 2021; Ma et al., 2022). These techniques have emerged as promising approaches in different fields of medicine. Specifically, convolutional neural networks, which are DL models, are adept at visual recognition and natural language processing and can be used to construct automated image analysis models for recognizing X-ray or MRI data (Bacanin et al., 2022; Zivkovic et al., 2022). Several ML algorithms, including eXtreme Gradient Boosting (XGBoost), Adaptive Boosting (AdaBoost), and gradient-boosted regression tree (GBRT), have been proven to be useful for predicting drug concentration (Ma et al., 2022). For instance, XGBoost was used to establish a model to predict the concentration of tacrolimus in patients with autoimmune diseases and a model to predict the active moiety concentration of risperidone based on initial TDM. CatBoost was used to develop a model to predict the concentration of quetiapine in patients with schizophrenia and depression, and an ensemble model using five algorithms (XGBoost, GBRT, Bagging, ExtraTree, and decision tree) was applied to predict the concentration of vancomycin in children (Huang et al., 2021; Zheng et al., 2021; Hao et al., 2023). Additionally, in response to theoretical and practical global optimization problems, SI techniques are very popular for the model optimization of ML and DL algorithms (Bacanin et al., 2021; Zivkovic et al., 2022). With the larger sample size of input data, these AI models can be continually optimized to achieve better performance and practicality.

In this study, we aimed to explore the factors influencing sertraline concentration and develop a prediction model for sertraline concentration through AI techniques. We pursued to facilitate the rational sertraline regimen at an individualized level and provide a reference for other antidepressant drug doses or for concentration prediction through the combination of medicine and AI techniques.

## Materials and methods

### Participants and study design

**FIGURE 1:**
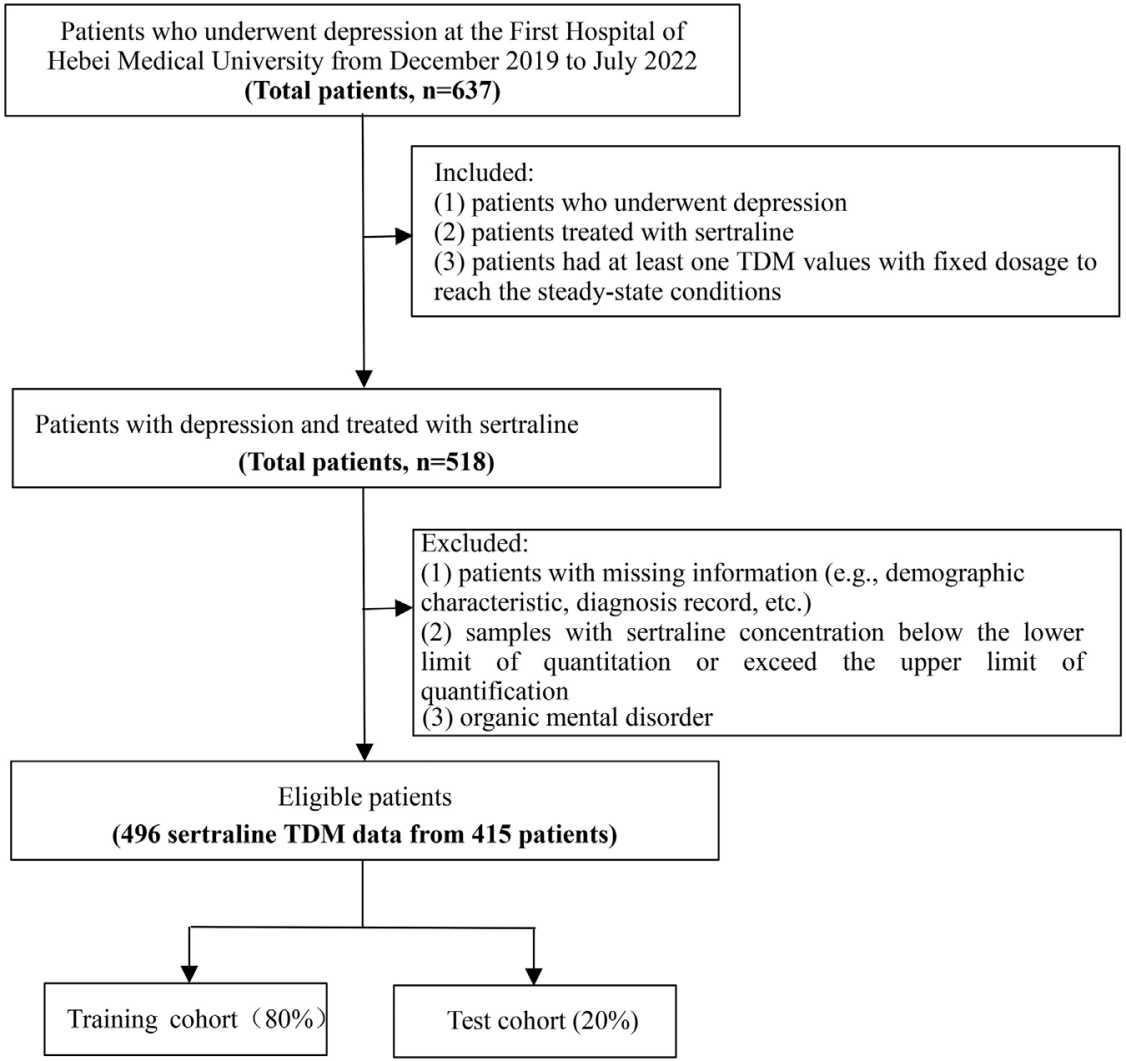
Workflow of sample selection.

This retrospective study was conducted from January 2020 to December 2021 at the First Hospital of Hebei Medical University, and data from inpatients with depression receiving routine clinical treatment with sertraline were analyzed. Patients enrolled in the study had two TDM values. One was the initial TDM value, which was measured for at least 5–7 days with a fixed sertraline dose to reach steady-state conditions. The other was the value measured closest to the initial TDM. The inclusion criteria were as follows: (1) patients diagnosed with depression from discharge records; (2) patients treated with sertraline; and (3) patients with at least one TDM value given at a fixed dosage to reach the steady-state conditions. The exclusion criteria were as follows: (1) patients with missing information (such as demographic characteristics and diagnosis records); (2) patients with sertraline concentrations less than the lower limit of quantitation or exceeding the upper limit of quantification; and (3) patients with organic mental disorder. The information assessed in the study included demographic information, the use of sertraline, combined medication, and biochemical indices. The workflow of sample selection is illustrated in Figure 1.

### Sertraline assay

The plasma sertraline concentration was measured via UPLC–MS/MS (Waters ACQUITYI-X, Waters Corp., Milford, United States of America). The sample was separated on a Waters ACQUITY UPLC BEH C18 column (2.1 mm × 50 mm, i.d.; 1.7 μm, Waters, Milford, CT, United States of America) and eluted with a step gradient mobile phase consisting of 0.1% formic water (solvent A) and methanol (solvent B): 0–0.5 min, 20% B; 0.5–0.6 min, 20–45% B; 0.6–1.8 min, 45–80% B; 1.8–1.9 min, 80–95% B; 1.9–2.5 min, 95% B; 2.5–2.6 min, 95–20% B; and 2.6–3.2 min, 20% B. The flow rate was 0.4 mL/min, and the injection volume was 5 μL. Mass spectrometric detection was performed via electrospray ionization in the positive ion multiple reaction monitoring mode. The transitions of the precursor ion and the production of the analyte were m/z 305.97→158.97 for sertraline and m/z 309.00→158.86 for sertraline-d3. The linear range for sertraline was 2.5–320 ng/mL (correlation coefficient *R* ^2^ = 0.9999). Both the intra- and inter-day precision and accuracy were within 15%.

### Variable selection

Multiple variables may influence sertraline concentration, including demographic data (age, sex, weight, and height), sertraline information (dosage and concentration), drug combinations (CYP2B6/CYP2C19 inhibitors, CYP2B6/CYP2C19 competitive inhibitor, and drugs with high plasma protein-binding rates), and data of assay indices (renal function, liver function, prolactin [PRL], and routine blood test results).

**FIGURE 2:**
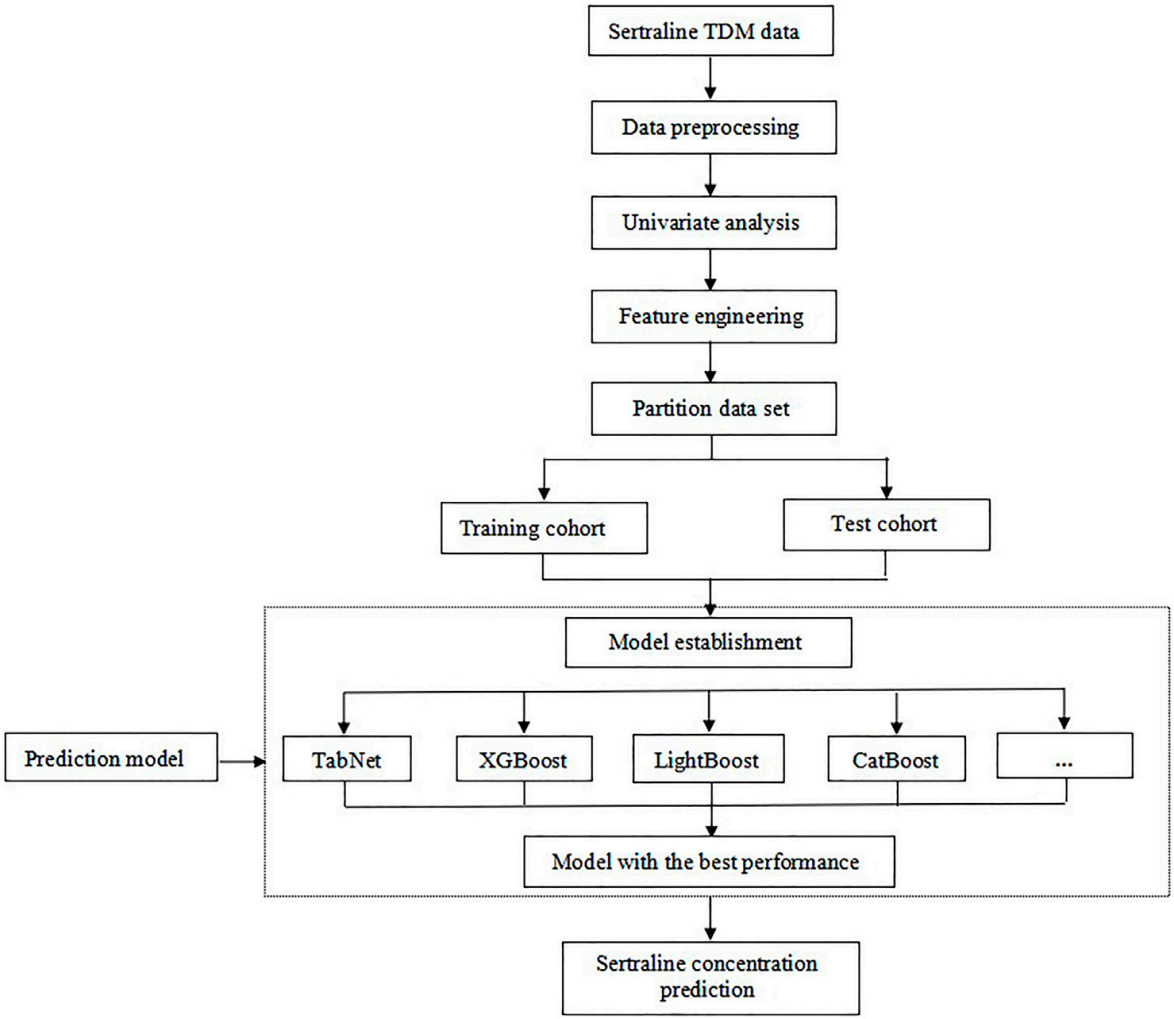
Workflow of data processing and modeling.

The workflow of the data analysis is illustrated in Figure 2. First, univariate analysis was performed on all the data to screen for significant variables, and *p* < 0.05 was considered to indicate statistical significance. After that, the sequential forward selection (SFS) algorithm was applied for feature engineering to select the minimum size and optimum performance of the feature subset. The algorithm starts the search from an empty set, and a feature is added to this feature subset at a time. Once the evaluation index *R* ^2^ reaches the optimal value, iteration is stopped. The feature subset of the previous round was considered the optimal feature selection result.

### Model establishment

According to the 8:2 proportional division, the total study population was divided into training and testing cohorts. As depicted in Figure 1, nine different algorithms were used for modeling to compare the ability of various models to predict sertraline concentrations; these included XGBoost, LightGBM, CatBoost, random forest (RF), gradient-boosted decision tree (GBDT), support vector machine (SVM), lasso regression (LR), artificial neural network (ANN), and TabNet algorithms. To measure and compare model performance, *R* ^2^, mean square error (MSE), root mean square error (RMSE), and mean absolute error (MAE) were used as metrics. The calculating formula is as follows:

$${\text{MSE}=}^{\frac{1}{n}\sum_{i=1}^{n}{\left(y_{i}-{\hat{y}}_{i}\right)}^{2}},$$

$${\text{RMSE}=}^{\sqrt{\frac{1}{n}\sum_{i=1}^{n}{\left(y_{i}-{\hat{y}}_{i}\right)}^{2}}},$$

$${\text{MAE}=}^{\frac{1}{n}\sum_{i=1}^{n}\frac{1}{n}\left|\left(y_{i}-{\hat{y}}_{i}\right)\right|},$$

$$R^{2}=1-\frac{MSE\left(\hat{y,}y\right)}{Var\left(y\right)},$$

where n is the number of samples; $y_{i}$ is the true value; and $\overset{i}{\overset{\wedge }{y}}$ is the predicted value. *R* ^2^ represents the goodness of fit of the model, and the value range is 0–1. The larger value indicates the better fit of the model. In terms of the MSE, RMSE, and MAE, when their values decrease, the model has better fitting goodness. Additionally, the accuracy of the predicted concentration within ±30% of the actual concentration was used for evaluating predictive performance. The algorithm with the highest metric values was selected for the prediction model of sertraline concentration.

The importance scores of the variables were calculated and ranked using the algorithm with the best predictive performance. Variables with higher importance scores were more closely related to the accurate prediction of sertraline concentration. Then, SHapley Additive exPlanations (SHAP) were conducted to visually interpret the positive or negative impacts of important variables on the model output.

### Statistical analysis

Statistical analysis was performed using the Statistical Package for the Social Sciences software version 23.0 and Python 3.9.12. The continuous non-normally distributed variable was expressed as the median and interquartile range (IQR, 25th and 75th percentiles) and was compared by Spearman’s correlation test, as appropriate. For binary variables, the Mann–Whitney U test was used for analysis. The categorical variable was presented as the number and percentage (%) and was analyzed by the chi-squared test.

## Results

### Baseline information

From December 2019 to July 2022, at the First Hospital of Hebei Medical University, a total of 415 eligible patients were enrolled in this study, with 496 samples from whom the sertraline concentration was measured. The baseline information of the study population is shown in Table 1. It can be seen that the median (IQR) age of the patients was 16 (14.00–27.00) years, the median (IQR) weight of the patients was 56.67 (50.33–68.00) kg, the median (IQR) height was 163.00 (158.00–169.00) cm, and males were 27.42% of the total. The median (IQR) value of sertraline concentration was 59.00 (34.04–85.56) ng/mL, and the median (IQR) dose of sertraline was 100 (100–150) mg. The percentage of patients using combination therapies was 0.81% for the CYP2B6 inhibitor (clopidogrel and voriconazole), 0.81% for the CYP2B6 competitive inhibitor (methadone, fluoxetine, and disulfiram), 3.63% for the CYP2C19 inhibitor (esomeprazole, fluvoxamine, voriconazole, and omeprazole), and 9.27% for the CYP2C19 competitive inhibitor (citalopram, escitalopram, clomipramine, clozapine, venlafaxine, diazepam, doxepin, and fluoxetine). The median (IQR) α-hydroxybutyrate dehydrogenase level was 101.00 (90.30–117.00) U/L, the median (IQR) alanine aminotransferase (ALT) level was 15.60 (10.70–24.60) U/L, the median (IQR) aspartate transaminase (AST) level was 19.30 (15.90–23.50) U/L, and the median (IQR) uric acid (UA) level was 287.75 (235.43–346.52) μmol/L.

**TABLE 1:** Description of demographic and clinical characteristics.

Category	Variable	Median (IQR)| n (%)	Miss rate (%)	*p*
Sertraline information	Concentration, median (IQR)	59.00 (34.04–85.56)	0.00	
	Dose, median (IQR)		0.00	<0.001
	50	111(22.38%)		
	100	145 (29.23%)		
	150	170 (34.27%)		
	200	70 (14.11%)		
Demographic information	Age, median (IQR)	16.00 (14.00–27.00)	0.00	0.002
	Sex, n (%)		0.00	<0.001
	Female	360 (72.58%)		
	Male	136 (27.42%)		
	Weight, median (IQR)	56.67 (50.33–68.00)	71.57	0.06
	Height, median (IQR)	163.00 (158.00–169.00)	72.78	0.107
Combination	CYP2B6 inhibitor, n (%)	4 (0.81%)	0.00	0.976
	CYP2B6 competitive inhibitor, n (%)	4 (0.81%)	0.00	0.767
	CYP2C19 inhibitor, n (%)	18 (3.63%)	0.00	0.714
	CYP2C19 competitive inhibitor, n (%)	46 (9.27%)	0.00	0.6
	Drugs with high plasma protein binding rate, n (%)	1(0.20%)	0.00	1
Laboratory parameters	AFU (IQR)	17.70 (13.80–21.10)	9.68	0.032
	HBDH, median (IQR)	101.00 (90.30–117.00)	22.18	<0.001
	GGT, median (IQR)	15.00 (12.00–23.00)	21.57	0.813
	ALT, median (IQR)	15.60(10.70–24.60)	21.57	0.008
	LDH, median (IQR)	161.00 (140.00–183.00)	22.18	<0.001
	AST, median (IQR)	19.30 (15.90–23.50)	21.57	0.001
	Urea, median (IQR)	3.97 (3.31–4.83)	4.03	0.421
	UA, median (IQR)	287.75 (235.43–346.52)	4.03	<0.001
	TP, median (IQR)	65.80 (63.60–69.05)	1.21	0.479
	WBC, median (IQR)	6.00 (5.10–7.30)	4.03	0.32
	AL, median (IQR)	40.90 (39.00–43.58)	1.21	0.292
	HCT, median (IQR)	36.80 (31.30–39.60)	6.85	0.457
	RBC, median (IQR)	4.34 (4.03–4.67)	4.03	0.333
	Cr, median (IQR)	55.75 (49.30–63.00)	4.03	0.379
	CK, median (IQR)	68.00 (51.00–99.75)	22.18	0.235
	PC, median (IQR)	252.00 (213.00–293.00)	4.03	0.11
	HB, median (IQR)	127.50 (118.00–138.00)	4.03	0.018
	NEU%, median (IQR)	51.80 (39.90–59.70)	4.03	0.003
	ANC, median (IQR)	3.30 (2.60–4.10)	4.03	0.03
	CO_2_CP, median (IQR)	26.00 (24.00–27.00)	3.43	0.004
	MONO%, median (IQR)	7.20 (5.40–8.60)	4.03	0.16
	MON, median (IQR)	0.50 (0.40–0.58)	4.03	0.054
	BASO%, median (IQR)	0.40 (0.30–0.60)	28.43	0.496
	BASO, median (IQR)	0.00 (0.00–0.01)	28.43	0.899
	ESO%, median (IQR)	2.10 (1.50–3.00)	28.43	0.085
	AEC, median (IQR)	0.10 (0.10–0.20)	28.43	0.203
	ALT/AST, median (IQR)	1.23 (0.92–1.55)	25.81	0.272
	MCV, median (IQR)	88.20 (84.80–91.57)	6.85	0.446
	MPV, median (IQR)	8.40 (7.70–9.20)	4.03	0.535
	MCHC, median (IQR)	335.00 (326.00–343.00)	4.03	0.037
	MCH, median (IQR)	29.70 (28.38–30.90)	4.03	0.137
	TBA, median (IQR)	3.20 (1.90–4.90)	1.21	0.367
	TBil, median (IQR)	8.40 (6.50–11.00)	1.21	0.012
	Lym%, median (IQR)	31.25(22.48–39.50)	4.03	0.315
	ALC, median (IQR)	2.00 (1.68–2.50)	4.03	0.23
	GLOB, median (IQR)	24.80 (22.80–27.48)	1.21	0.991
	A/G, median (IQR)	1.63 (1.47–1.83)	1.21	0.796
	DB, median (IQR)	1.70 (1.30–2.30)	1.21	0.003
	RDW CV, median (IQR)	13.30 (12.90–14.30)	28.43	0.01
	RDW SD, median (IQR)	42.40 (40.70–44.60)	35.89	0.403
	CHE, median (IQR)	7,124.00 (6,263.00–7,947.75)	1.21	0.121
	ADD, median (IQR)	10.80 (8.78–12.90)	9.68	0.001
	PDW, median (IQR)	16.40 (16.10–16.70)	28.43	0.878
	PCT, median (IQR)	0.21 (0.18–0.25)	6.85	0.151
	SIB, median (IQR)	6.75 (5.10–8.70)	1.21	0.023

IQR, interquartile range; AFU, a-L-fucosidase; HBDH, α- hydroxybutyrate dehydrogenase; GGT, γ-glutamyl transpeptidase; ALT, alanine aminotransferase; LDH, lactic dehydrogenase; AST, aspartate transaminase; UA, uric acid; TP, total protein; WBC, white blood cell count; RBC, red blood cell count; Cr, creatinine; CK, creatine kinase; AL, albumin; HCT, hematocrit; PC, platelet count; HB, hemoglobin; NEU%, neutrophil percentage; ANC, absolute neutrophil count; CO_2_CP, carbon dioxide combining power; MONO%, monocyte percentage; MON, monocyte absolute count; BASO%, basophil percentage; BASO, basophil absolute count; ESO%, percentage of eosinophils; AEC, absolute eosinophil count; MCV, mean corpuscular volume; MPV, mean platelet volume; MCHC, mean cell hemoglobin concentration; MCH, mean cell hemoglobin; TBA, total bile acid; TBil, total bilirubin; Lym%, lymphocyte percentage; ALC, absolute lymphocyte count; GLOB, globulin; A/G, albumin–globulin ratio; DB, direct bilirubin; RDW, red blood cell volume distribution width; CHE, cholinesterase; ADD, adenosine deaminase; PDW, platelet distribution width; PCT, plateletcrit; SIB, serum indirect bilirubin.

### Variable selection

In the univariate analysis, several variables were excluded because of an extremely uneven distribution or lots of missing values. The statistical results for the remaining 55 variables are shown in Table 1. Among them, 20 significant variables, including dose, sex, age, weight, AFU, HBDH, and ALT, had *p* < 0.05.

**FIGURE 3:**
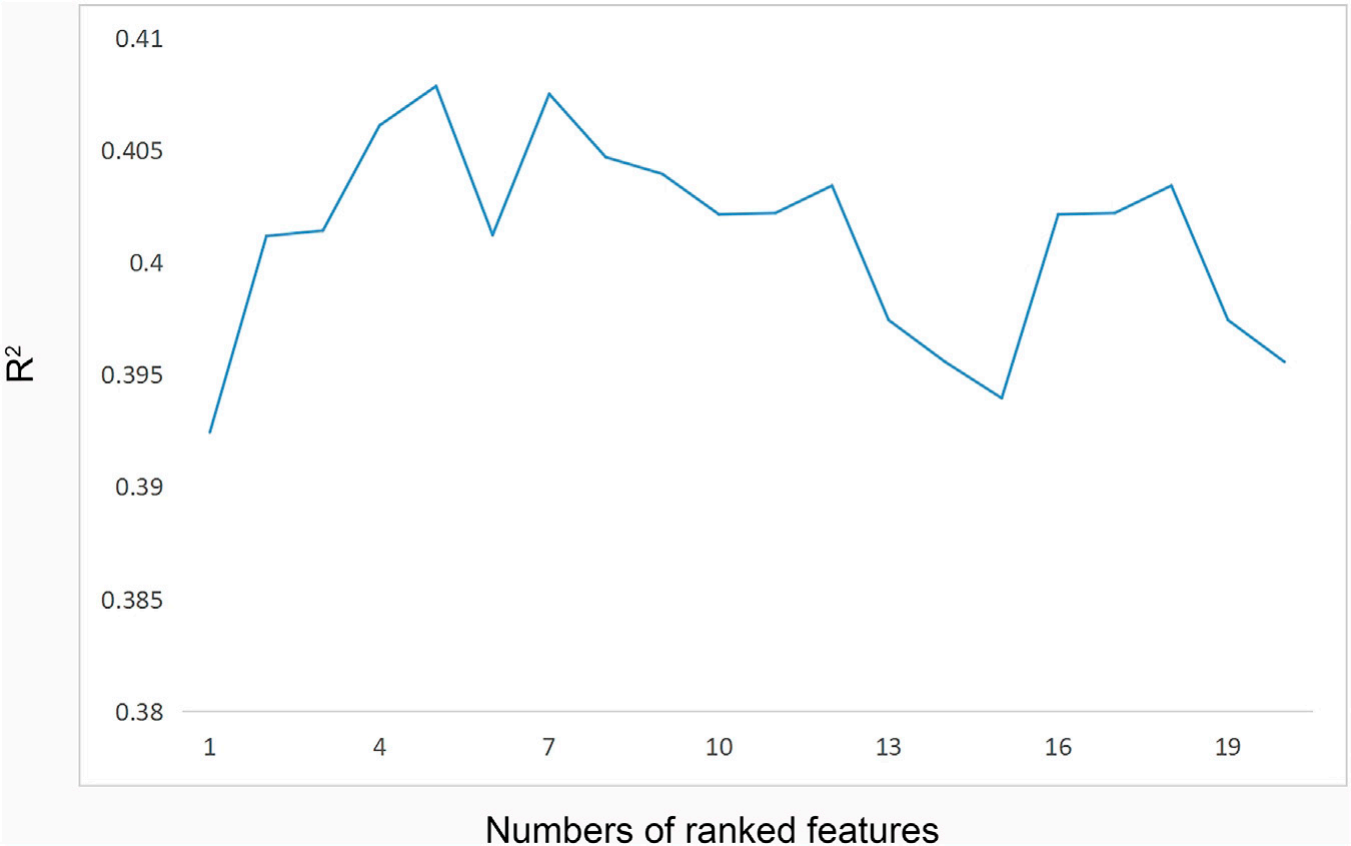
*R* ^2^ of the CatBoost model corresponding to the number of ranked features.

CatBoost models were established using the selected 1 to 20 variables, and the *R* ^2^ of each model was obtained (Figure 3). With an increasing number of included variables, the *R* ^2^ value continued to increase, reached its maximum value when five variables were selected (*R* ^2^ = 0.408), and then, decreased. As we pursued a concise and accurate model with minimal variables but the highest predictive performance, the first five important variables were selected to establish the personalized medication model: daily dose of sertraline, ALT, sex, AST and UA.

### Model performance and interpretation

**FIGURE 4:**
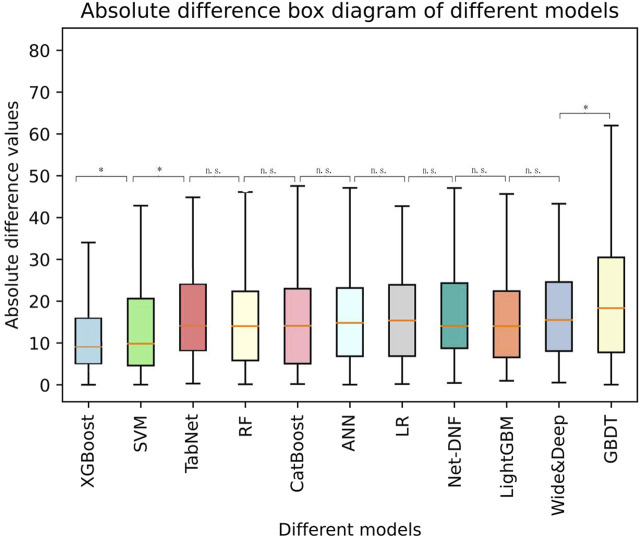
A boxplot of the absolute difference in *R* ^2^ of different models tested by the Wilcoxon signed-rank test. *: significant; ns: not significant.

Based on the selected variables, nine algorithm models (XGBoost, LightGBM, CatBoost, RF, GBDT, SVM, LR, ANN, and TabNet) for predicting sertraline concentration were established. The final model performance in the testing cohort (N = 332) is illustrated in Table 2. In addition, a boxplot of all the models is shown in Figure 4, which illustrates the absolute difference in *R* ^2^ between two models tested by the Wilcoxon signed-rank test after the *R* ^2^ was ranked from the highest to lowest. The XGBoost model had the highest *R* ^2^ (0.63), which was significantly greater than that of the other models (*p* < 0.05), demonstrating a moderate model fit. In addition to this, in the XGBoost model, the accuracy of the predicted concentration within ±30% of the actual concentration was 60.00%, the highest of the nine models. The MAE and RMSE of the XGBoost model were 15.35 and 20.06, respectively, and these low values represent a good model fit. Thus, XGBoost had the most prominent model performance and was chosen to be applied for the prediction model.

**TABLE 2:** Prediction results of the nine algorithms models in the testing cohort.

Model	*R* ^2^	RMSE	MAE	Accuracy within ±30 (%) range
XGBoost	0.63	20.06	15.35	60.00
LightGBM	0.55	22.02	16.33	58.00
CatBoost	0.59	20.95	16.34	56.00
RF	0.60	20.66	16.00	55.00
GBDT	0.33	26.78	19.55	54.00
SVM	0.61	20.58	15.20	54.00
LR	0.56	21.62	17.30	51.00
ANN	0.57	21.56	17.16	54.00
TabNet	0.61	20.47	16.16	54.00
Wide&Deep	0.51	22.89	18.07	52.00
Net-DNF	0.56	21.75	17.46	53.00

RF, random forest; GBDT, gradient-boosted decision tree; SVM, support vector machine; LR, lasso regression; ANN, artificial neural network; MSE, mean square error; RMSE, root mean square error; MAE, mean absolute error.

On this basis, the importance scores of five selected variables were calculated and ranked by XGBoost (Table 3). Specifically, the importance score of the sertraline dose (importance score = 0.629) was markedly greater than that of the other four variables, followed by AST (importance score = 0.109), UA (importance score = 0.1), sex (importance score = 0.082), and ALT (importance score = 0.08). A higher importance score indicates a greater impact of this variable on the prediction of sertraline concentration.

**TABLE 3:** Importance scores of the variables in the XGBoost model.

Variable	Importance
Dose	0.629
AST	0.109
UA	0.1
Sex	0.082
ALT	0.08

ALT, alanine aminotransferase; AST, aspartate transaminase; UA, uric acid.

**FIGURE 5:**
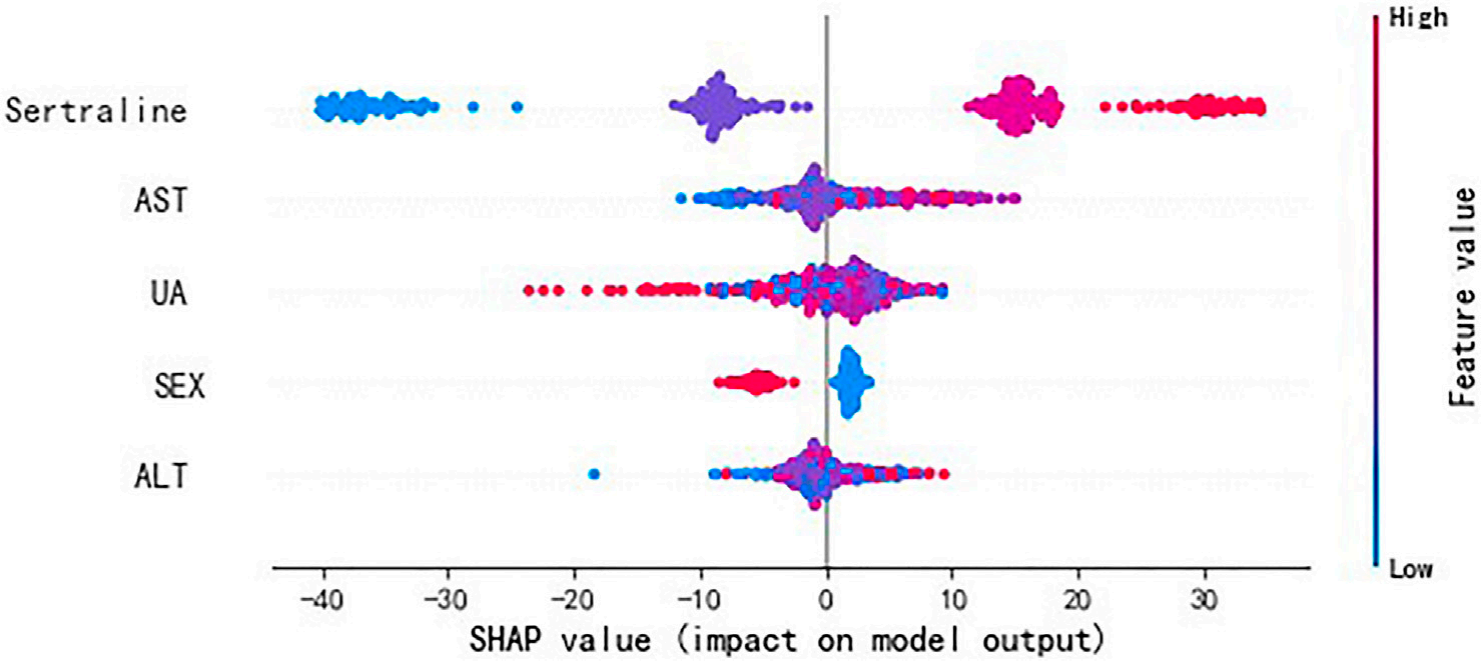
SHAP values of important variables. The dot color is redder when the feature value gets higher and bluer when the feature value gets lower. SHAP value means the impact of the variable on the model output.

For the visualization of the variable importance, we used SHAP to quantify the magnitude and direction (positive or negative) of the variable’s influence on sertraline concentration (Figure 5). The feature value (represented by the dot color) represents the contribution of each variable to the predictive power of the model and was ranked according to the importance. A larger width of the color area indicates a greater impact of the variable. Consistent with the results of XGBoost, the variables in the descending order of effect on sertraline concentration were sertraline dose, AST, UA, sex, and ALT. In terms of the binary variable “sex,” “0” corresponds to females and “1” corresponds to males. For the daily dose of sertraline and for ALT and AST, the dot color is redder (feature value is greater) when the SHAP value becomes larger, while it is bluer (feature value is lower) when the SHAP value becomes smaller, thus revealing the positive impacts of these variables on sertraline concentration. However, UA was negatively correlated with the model prediction outcome. In terms of sex, as a binary variable, male patients had a negative correlation with sertraline concentration, and female patients had a positive correlation with sertraline concentration.

The testing cohort consisted of 83 patients. The predicted value of sertraline concentration was compared with the true value in the testing cohort. Finally, the proportion of the predicted sertraline blood concentration within ±30% of the true value was calculated. The results are illustrated in Table 4. The prediction accuracy of the drug concentration in the therapeutic window (10–150 ng/mL) was 62.5%.

**TABLE 4:** Prediction accuracy of the XGBoost model.

Blood concentration	Accuracy	Cases
<10 ng/mL	0	4
10–150 ng/mL	62.5%	96
>150 ng/mL	0	0

## Discussion

Our study focused on the establishment of a personalized medication model for the prediction of sertraline concentration in patients with depression. In our study, nine ML models were established. By comparison, for XGBoost, the model with the most accurate prediction performance was selected with an *R* ^2^ of 0.63. XGBoost is an ML algorithm that is a leading-edge method based on the decision tree principle and the effective upgrade of the GBDT algorithm (Yan et al., 2022). It integrates a series of decision trees to achieve the classification or regression goals (Li et al., 2019). ML methods are suitable for processing large volumes of real-world data, addressing missing values and high-dimensional data, and capturing complicated relationships between variables, especially for retrospective studies (Lee et al., 2022; Matsuzaki et al., 2022). Compared with those of other ML models, our study showed better performance (the accuracy of the predicted concentration within ±30% of the actual concentration was 62.5%) in the testing cohort and a larger sample size, which led to a more mature model for the application and reference (Huang et al., 2021). Sertraline is one of the most commonly prescribed antidepressants in China. There are large individual differences in sertraline plasma concentration. Therefore, it is meaningful to establish a model to predict the concentration of sertraline.

The results of our study revealed that dose is the most important variable affecting the plasma concentration of sertraline and that there is a positive relationship between the daily dose of sertraline and plasma concentration. This finding is consistent with the pharmacokinetics of sertraline and previously described associations (Tini et al., 2022). A study of the population pharmacokinetics of sertraline in healthy subjects also showed that following multiple oral doses, the C_max_ and AUC∞ increased proportionally with dose across the entire dose range (5–200 mg), while the bioavailability, T_max_, and t_1/2_ remained constant with dose (Alhadab and Brundage, 2020).

Sertraline is metabolized mainly in the liver. Hepatic metabolism is thought to play a major role in the overall clearance of sertraline (Zhou et al., 2019). *In vivo*, the levels of ALT and AST can reflect the liver function of patients. High ALT and AST levels indicate that patients may have impaired liver function, which may inhibit the metabolism of sertraline in the liver, resulting in a high plasma sertraline concentration.

The excretion and secretion of UA are mediated by multiple transporters in the kidney. The level of UA increases once the function of the kidney is impaired (Okui et al., 2020). There have been few studies on the correlation between UA levels and sertraline plasma concentrations, but our study revealed a negative correlation between them. Abnormal renal function may lead to a reduction in the reabsorption of UA in the kidney, resulting in a decrease in UA levels in the body. Sertraline and its metabolites are mainly excreted in feces and urine. Renal dysfunction may inhibit the renal excretion of sertraline, resulting in a high plasma concentration of sertraline. This may explain why UA was negatively correlated with the sertraline concentration.

Sex is another important variable that contributes to the plasma level of sertraline. Several studies have suggested that the apparent clearance of sertraline is significantly increased in male patients and that there is an age/sex interaction, which indicates a low plasma concentration of sertraline in male patients (Davies et al., 2016). Similarly, our study displayed a negative correlation between males and sertraline plasma concentration. The mechanistic processes underlying sex-specific pharmacokinetics can be divided into physiological and molecular factors. First, sertraline and desmethylsertraline showed high affinity for P-gp, which meditates the absorption of sertraline in the small intestine. A study confirmed that, compared with female rats, male rats exhibited higher relative P-gp expression in the intestine, which may result in increased intestinal efflux of sertraline in males (Mai et al., 2018). Second, sex-related pharmacokinetic differences include the generally lower bodyweight and organ size, a greater percentage of body fat, a lower glomerular filtration rate, and different gastric motility in females compared to males, which may explain low sertraline concentration in males (Li et al., 2020).

Previous studies indicated that concomitant treatment with an inhibitor of CYP2B6/2C19 may influence the concentration of sertraline (Preskorn et al., 2007; Gjestad et al., 2015). However, in our study, there was no correlation between the use of combined medication and the plasma concentration of sertraline. This may be due to the small number of patients treated with the combination therapy.

One advantage of this study is that we conducted subgroup analysis based on different concentration ranges to determine the respective prediction performance at diverse concentrations, helping supplement the data for the specified concentration range to refine the model continuously. In addition, we enhanced the model performance by leveraging the capabilities of ML. Through the stratified mining of data, ML methods can recognize and analyze multiple influencing factors in the real world. In particular, XGBoost has the ability to deal with data rapidly and effectively, reduce model overfitting, and process clinical data with a mass of outliers and missing values to construct an accurate prediction model. The combination of ML and personalized medicine has improved the effectiveness of precision medicine in clinical practice. Finally, to the best of our knowledge, our study is the first to use ML techniques to predict sertraline concentration in patients with depression using ML techniques. This study fills the gap in this field and provides a reference for the rational clinical use of sertraline.

However, there are several limitations in our study. First, this was a retrospective study using real-world data rather than a randomized controlled trial, inevitably resulting in some biases. In future studies, we intend to employ a randomized controlled trial utilizing stringent inclusion criteria to effectively manage potential confounding factors that might influence patient outcomes. Following the principle of randomization, subjects are allocated to each group with equal probability. This ensures that potential confounding variables are evenly distributed among the groups. Second, since TDM tests of sertraline were only conducted beginning in November 2019 at the First Hospital of Hebei Medical University, the final sample size was limited, which could lead to inaccurate findings in this study. Expanding the sample size or conducting prospective research in multiple centers is what we should strive for in the future. Third, data for some variables, such as height and weight, were deleted due to a high missing data rate or imbalanced sample size. In future studies, it is necessary to strengthen the training of doctors in recording and collecting data. We will also apply additional methods, such as interpolation, to process missing value to solve this problem. Notably, the theoretical shortcomings of ML models cannot be ignored. ML models suffer from overfitting, especially those with modest datasets (Bacanin et al., 2021). Dropout estimation by SI algorithms can help solve this issue (Bacanin et al., 2021). Previous studies have shown good results by using hybrid methods of SI and ML (Basha et al., 2021; Wainer and Fonseca, 2021; Zivkovic et al., 2021). In the future, we will pursue a more optimized model for predicting the sertraline concentration.

## Conclusion

In conclusion, in this study, nine different AI algorithms were used for modeling to compare the ability of the models to predict sertraline concentration, and XGBoost was chosen to establish the personalized medication model with the best performance. Five important variables were found through ML to be correlated with the sertraline concentration. The personalized medication model of sertraline for patients with depression based on XGBoost had an acceptable prediction ability, which can be improved with a larger sample size and provide a reference for clinicians to propose the optimal medication regimen.

## Data Availability

The raw data supporting the conclusion of this article will be made available by the authors, without undue reservation.
